# PANoptosis: a new perspective for targeting programmed cell death after spinal cord injury

**DOI:** 10.3389/fimmu.2026.1772287

**Published:** 2026-02-20

**Authors:** Qiheng Qian, Lei Shi, Jiding Xie, Xinyu Zhao, Xiangqi Meng

**Affiliations:** 1 Orthopedics, Suzhou Hospital of Traditional Chinese Medicine, Suzhou, Jiangsu, China; 2 College of Physical Education, Yangzhou University, Yangzhou, Jiangsu, China; 3 Experimental Research Center, China Academy of Chinese Medical Sciences, Beijing, China; 4 Pei County Hospital of Traditional Chinese Medicine, Xuzhou, Jiangsu, China

**Keywords:** inflammation, PANoptosis, pyroptosis, spinal cord injury, Z-DNA binding protein 1 (ZBP1)

## Abstract

Spinal cord injury (SCI) is a devastating condition characterized by a complex cascade of secondary injury following an initial mechanical insult. Among the mechanisms of secondary injury, multiple forms of programmed cell death (PCD)—including apoptosis, necroptosis, and pyroptosis—play key roles in tissue degeneration and loss of function. PANoptosis is a recently proposed concept of cell death that integrates the aforementioned three pro-inflammatory PCD pathways into a single process via a multi-protein complex called the PANoptosome. This review summarizes evidence for the involvement of PANoptosis in SCI pathophysiology and discusses intervention strategies targeting PANoptosis. Targeting PANoptosis offers a novel, holistic approach to mitigating secondary injury in SCI. However, its clinical translation remains challenging and requires further studies to validate efficacy and safety. Elucidating the assembly mechanisms of the PANoptosome and identifying critical regulatory molecules will aid in developing combination therapies and biomarkers to improve neuronal survival and functional recovery in patients with SCI.

## 1 Introduction

Spinal cord injury (SCI) is a severe central nervous system injury that can lead to loss of motor, sensory, and autonomic function, imposing a heavy burden on patients and society (1). The pathophysiology of SCI involves two phases: an initial primary injury and a subsequent secondary injury. Primary injury causes immediate and often irreversible structural damage at the moment of trauma. Secondary injury unfolds over hours to weeks post-injury and is driven by biochemical cascades—including local ischemia, oxidative stress, inflammatory responses, and excitotoxicity—that further damage the normal tissue surrounding the initial lesion (2). A key feature of secondary injury is extensive programmed cell death (PCD) in neurons and glial cells, which exacerbates tissue damage and neurological dysfunction (3). Early studies found that in the region of secondary injury after SCI, many neurons and oligodendrocytes underwent apoptosis, characterized by DNA fragmentation and caspase-3 activation (4). Subsequently, researchers discovered that necroptosis and pyroptosis also contribute to SCI pathology (5, 6). After SCI, these different cell death pathways often occur concurrently. They are thought to collectively drive secondary tissue damage and functional decline.

Despite numerous experimental studies targeting individual PCD pathways showing some efficacy, there is still a lack of effective therapies to significantly lessen secondary damage after SCI (7). Notably, these PCD pathways are not independent; on the contrary, they interact and can compensate for one another. In various disease models, inhibiting one cell death pathway may prompt cells to switch to an alternative death pathway (8, 9). This “see-saw” relationship led to the concept of “PANoptosis,” which regards pyroptosis, apoptosis, and necroptosis as an interconnected, unified form of cell death (10). PANoptosis emphasizes the extensive crosstalk and coordination among these three PCD pathways, which is orchestrated by a multi-protein complex called the PANoptosome.

Recent studies suggest that PANoptosis may play an important role in SCI secondary injury. In animal models, activation markers of apoptosis, necroptosis, and pyroptosis can all be detected simultaneously in injured spinal cord tissue, suggesting that PANoptotic cell death (i.e. cell death with features of all three pathways) is one cause of the widespread cell loss in SCI (11, 12). These findings position PANoptosis as a new therapeutic target. By intervening in the PANoptosome or its key regulatory molecules, it may be possible to simultaneously inhibit apoptosis, necrosis, and pyroptosis. This approach could achieve more comprehensive neuroprotection than targeting any single pathway.

## 2 Assembly of the PANoptosome and its regulatory factors

PANoptosis is defined as an inflammatory programmed cell death pathway regulated by a multi-protein complex known as the PANoptosome, which coordinates the activation of pyroptosis, apoptosis, and necroptosis in a coupled manner (13, 14). The PANoptosome serves as a molecular platform or scaffold that brings together key signaling molecules from each of the three pathways, enabling cross-communication and the simultaneous execution of cell death programs (15). Foundational studies by Malireddi and colleagues first identified the PANoptosome complex in the context of innate immune responses to pathogens (10). PANoptosis is regulated by upstream receptors and molecular cascade signals that converge to assemble a polymeric signaling platform termed the PANoptosome (12). During signal transduction, the PANoptosome occupies a central integrative position, receiving and coordinating multiple upstream inputs to initiate downstream effector events (16). As a multiprotein complex, the PANoptosome typically forms following activation of innate immune sensors. Its components may include multiple caspases as well as RIPK1 and RIPK3 (15). Through this assembly, the complex not only promotes the activation of gasdermins and MLKL but also facilitates inflammasome assembly and activation (17). Through such assembly, the PANoptosome can concurrently activate executors such as caspase-1 (for pyroptosis), caspase-3/7 (for apoptosis), and MLKL via RIPK3 (for necroptosis) (18, 19). Cleaved caspase-1 (from inflammasome activation) induces gasdermin D-mediated membrane pores, causing pyroptosis. Meanwhile, caspase-8 and caspase-3 trigger DNA fragmentation (apoptosis), and phosphorylated MLKL compromises the plasma membrane (necroptosis) – all in the same dying cell (20, 21). PANoptosis thus represents a form of cell death that cannot be explained by any single pathway alone, highlighting the intricate interplay of PCD mechanisms.

Several key regulatory factors have been identified that modulate PANoptosome assembly and activity. Z-DNA binding protein 1 (ZBP1), also known as the DNA-dependent activator of interferon regulatory factors, functions as an innate nucleic acid sensor and immune receptor, and it plays a role in specific regions of the genome (22). Christgen et al. demonstrated a ZBP1-dependent PANoptosome that triggers pyroptosis, apoptosis, and necroptosis in macrophages during bacterial infection (23). A prototypical PANoptosome described in macrophages contains ZBP1 as a nucleic acid sensor, along with RIPK3, the inflammasome adaptor ASC, and caspase-8 (an apoptosis initiator), among other components (24, 25).

Among the constituent molecules, ZBP1 has emerged as a critical driver of PANoptosis in multiple contexts (26). Once activated, ZBP1 interacts with RIPK3 and other partners to nucleate PANoptosome formation. This nucleation instigates downstream caspase and kinase activation, leading to cell death. Caspase-8 is another pivotal component of many PANoptosomes. It acts as a molecular switch between apoptosis and necroptosis and also participates in inflammasome regulation (24). The dual roles of caspase-8 explain its inclusion in the PANoptotic complex – it can directly initiate apoptosis via caspase-3 and also modulate RIPK3/MLKL activity in necroptosis (27). Additionally, caspase-8 (when part of a PANoptosome) has been shown to license pyroptosis by promoting gasdermin processing in certain conditions (18).

Upstream regulators like cytokines and interferons can influence PANoptosis by inducing the expression of PANoptotic components. Interferon regulatory factor 1 (IRF1) was found to transcriptionally upregulate key PANoptosis mediators and promote PANoptosis as a tumor-suppressive mechanism (13, 28). Conversely, some molecules serve as negative regulators to restrain inappropriate PANoptosis. ADAR1, an RNA editing enzyme, can suppress PANoptosis by destabilizing ZBP1 transcripts or blocking ZBP1 sensing. This effect is demonstrated in cancer models where loss of ADAR1 unleashes ZBP1-driven cell death (29). These findings illustrate that the balance of PANoptotic signaling is under tight regulation by both positive and negative factors.

In summary, the assembly of the PANoptosome and the regulation of PANoptosis involve a network of interactions among sensors (e.g., ZBP1), adaptor/scaffold proteins (ASC, RIPK3, caspase-8, etc.), and regulatory factors (IRF1, ADAR1, etc.).

## 3 Mechanisms of PANoptosis in SCI

Emerging evidence suggests that PANoptosis plays a substantive role in the secondary injury mechanisms of spinal cord injury. In the injured spinal cord, the local microenvironment undergoes drastic changes. These include glutamate excitotoxicity, calcium influx, deprivation of trophic factors, and accumulation of reactive oxygen species (ROS), which together activate multiple cell death signaling pathways (30). As a result, apoptosis, necroptosis, and pyroptosis are often triggered in parallel in various cell types after SCI, ranging from neurons and oligodendrocytes to microglia and endothelial cells (31, 32). This section examines the mechanisms and evidence of PANoptosis in SCI and highlights how the three death modes converge in this context.

Concurrent activation of apoptosis, necroptosis, and pyroptosis after SCI: Extensive neuronal apoptosis has long been documented in the lesion penumbra following traumatic SCI, contributing to the loss of neurons and motor function (33, 34). Apoptotic cell death in SCI is mediated by both intrinsic (mitochondrial) and extrinsic (death receptor) pathways, such as injury-induced cytochrome c release and Fas receptor activation have been reported, leading to caspase-3 activation and DNA fragmentation in neurons and glia (35, 36). At the same time, necroptosis has been implicated in SCI by studies showing increased levels of RIPK1/RIPK3 signaling and MLKL phosphorylation in the spinal cord post-injury (37–39). Necroptotic cell death is pro-inflammatory due to plasma membrane rupture. It may especially affect oligodendrocytes and neurons that survive the initial insult but are later exposed to inflammatory cytokines (like TNF-α) in the lesion environment. Notably, inhibition of necroptosis through agents like necrostatin-1 (a RIPK1 kinase inhibitor) has been shown to reduce neuronal loss and improve functional recovery in rodent SCI models (40), underscoring necroptosis’ contribution to injury progression.

Simultaneously, pyroptosis – typically mediated by the NLRP3 inflammasome and caspase-1 – has emerged as another contributor to SCI pathophysiology (31). Following SCI, especially contusion or ischemia-reperfusion injury, there is often an upregulation of inflammasome components (NOD-like receptors, ASC) and the gasdermin family in the spinal cord (41, 42). For instance, NLRP3-dependent neuronal pyroptosis was observed in a spinal cord ischemia model, as evidenced by active caspase-1 and IL-1β release in neurons (43). The concurrent presence of TUNEL-positive apoptotic cells, RIPK3/MLKL-positive necroptotic cells, and gasdermin-positive pyroptotic cells in injured spinal cord tissue has led researchers to propose that PANoptosis—rather than any single mode of PCD—underlies the massive cell loss in SCI (44, 45). In other words, many cells in the injured spinal cord might be dying through a combined mechanism that features hallmarks of all three pathways (46). Apoptosis, necroptosis, and pyroptosis are interlinked contributors to secondary injury, each amplifying inflammatory and degenerative signals in the spinal cord milieu.

Molecular evidence of PANoptosis in SCI: Beyond histological markers, molecular and genetic studies have provided deeper evidence that PANoptotic mechanisms are at play in SCI. Transcriptomic analyses of SCI models have identified simultaneous changes in the expression of genes associated with apoptosis (e.g., Bax, Caspase-3), necroptosis (RIPK3, MLKL), and pyroptosis (NLRP3, GSDMD) after injury (47). In a pioneering bioinformatics study, Feng et al. (48) intersected differentially expressed genes (DEGs) from human SCI patient samples with a defined list of PANoptosis-related genes. Among these PANoptosis-related genes, some were known mediators, while others were less expected. Notably, BMX—a non-receptor tyrosine kinase—was identified as a crucial PANoptosis-related gene that is upregulated in SCI. Further network analysis indicated that BMX might regulate inflammatory cell death and immune responses after injury. This implicates novel factors like BMX in PANoptotic signaling and highlights potential cross-talk between cell death and immune activation in SCI. Another study applied machine-learning algorithms to identify hub PANoptosis-related genes in SCI. They discovered five key genes—CASP4, GSDMB, NAIP, NLRC4, and NLRP3—that consistently emerged as central nodes in SCI datasets (49). Interestingly, these genes include multiple inflammasome components (NLRC4 and NLRP3), an executor of pyroptosis (Gasdermin-B), an executioner caspase (caspase-4; in mice, this is analogous to caspase-11 for non-canonical pyroptosis), and an inflammasome sensor (NAIP). The identification of multiple inflammasome-related genes reinforces that pyroptotic signaling is deeply involved in SCI. It also suggests that therapeutic targeting of inflammasome components might confer neuroprotection by mitigating PANoptosis.

The most direct evidence of PANoptosis in SCI comes from *in vivo* experiments manipulating the PANoptosis pathway. A study on the TRIM56–YBX1–ZBP1 axis in SCI demonstrated that ZBP1-mediated PANoptosis indeed occurs in neurons after SCI (11). When ZBP1 expression was knocked down in injured mice, there was a marked reduction in the activation of caspase-1 (and the pyroptotic fragment gasdermin D N-terminal), cleaved caspase-3, and phosphorylated RIPK3/MLKL in spinal neurons. This indicates that ZBP1 is an upstream trigger whose presence is required for full engagement of the pyroptotic, apoptotic, and necroptotic machinery in SCI. Conversely, overexpression of YBX1 (which increases ZBP1) led to heightened activation of all three death pathways. In contrast, TRIM56 overexpression (which promotes YBX1 degradation) had the opposite effect, blunting these death signals. These findings confirm that the three PCD modes in SCI are mechanistically linked via common regulators, fitting the definition of PANoptosis. They also highlight how neuroinflammation and PANoptosis feed into each other. ZBP1 is known to be inducible by type I interferons during inflammation, and its activation of cell death can release damage-associated molecular patterns (DAMPs) that further stimulate inflammation. Thus, a cycle of inflammatory cell death can sustain secondary damage after SCI, with PANoptosis at its core.

**Figure 1:**
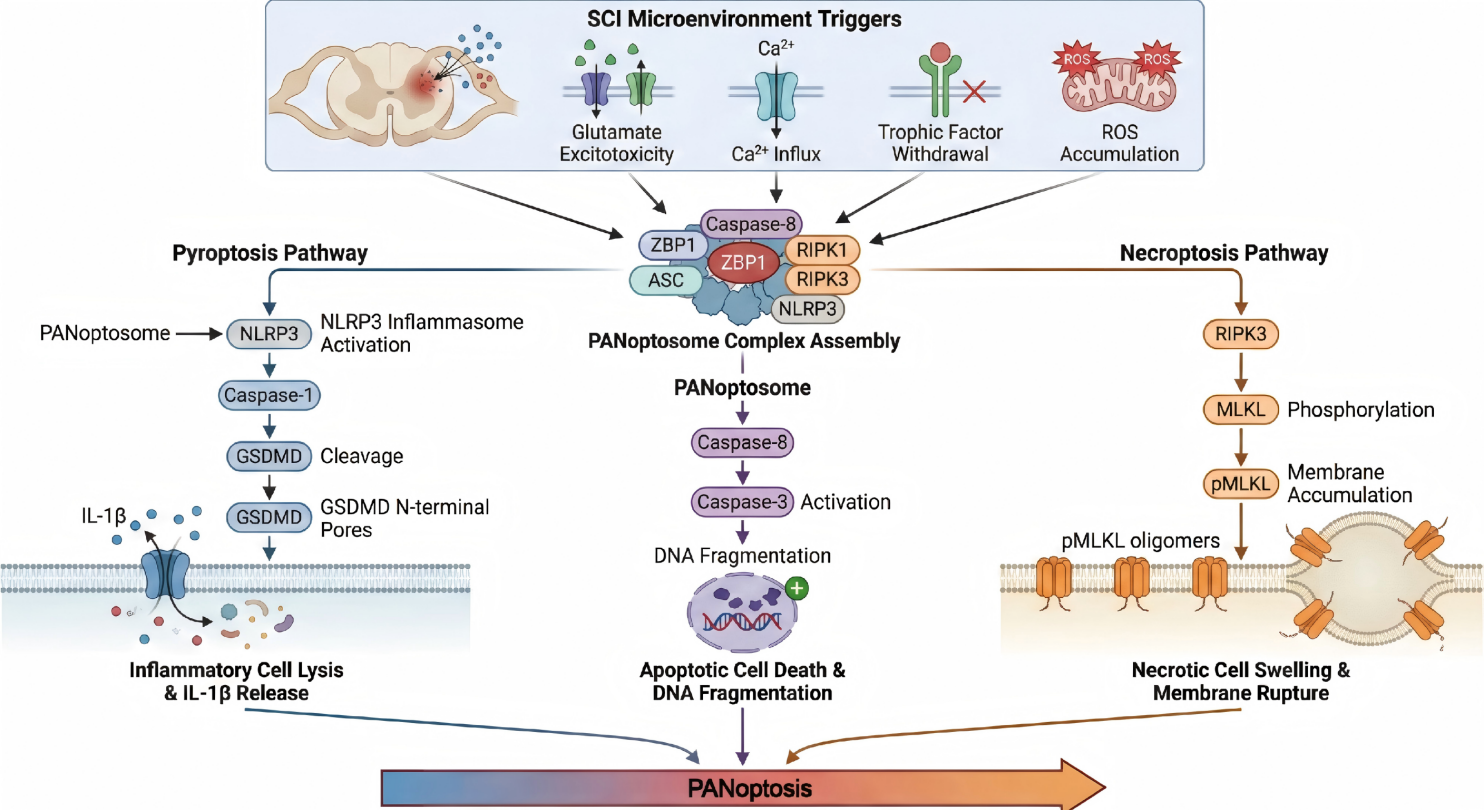
Mechanism of PANoptosis following spinal cord injury.

Other related forms of cell death: It is worth noting that SCI pathology is complex and involves additional forms of regulated cell death beyond PANoptosis. Ferroptosis (iron-dependent, lipid peroxidation-driven cell death) has been reported to occur in the subacute phase of SCI and contributes to oligodendrocyte and astrocyte death (47). Similarly, autophagy and parthanatos have been observed in experimental SCI (50). While these are distinct processes, they may intersect with PANoptosis pathways. For instance, excessive oxidative stress can simultaneously trigger ferroptosis and PANoptosis, or autophagic dysfunction might exacerbate inflammasome activation. Current evidence suggests PANoptosis is a dominant driver of cell death in the acute phase of SCI, but interplay with other cell death forms is an important subject for future research. Integrating PANoptosis with the broader “cell death landscape” of SCI will be necessary to fully understand secondary injury and to design combination therapies that address all destructive pathways (a schematic diagram of this mechanism is shown in **Figure 1**).

## 4 Potential targets and approaches for PANoptosis intervention to improve SCI outcomes

The recognition of PANoptosis as a form of cell death that integrates pyroptosis, apoptosis, and necroptosis provides a new perspective for understanding secondary injury in SCI (51). Traditional approaches to neuroprotection after SCI have largely focused on single pathways—for example, using caspase inhibitors to block apoptosis or using necroptosis inhibitors—yielding only partial benefits in experimental models (52). The PANoptosis paradigm suggests that these PCD pathways are so interconnected that silencing one may be insufficient, or even counterproductive, due to compensatory activation of the others. By contrast, targeting PANoptosis means intervening at points of convergence or common upstream triggers, thereby suppressing the entire network of inflammatory cell death.

A critical future challenge lies in identifying the optimal intervention targets within the PANoptosis pathway. While directly targeting core molecules such as ZBP1 or caspase-8 may offer broad control over PANoptosis, such approaches carry significant risks. These molecules perform essential physiological functions in host defense and tissue homeostasis (53). For instance, germline loss of Casp8 in mice causes mid-gestational embryonic lethality, highlighting the essential developmental functions of caspase-8 (54). Moreover, T cell–specific Casp8 deficiency leads to profound immune dysregulation and an age-dependent lethal lympho-infiltrative disorder. This underscores the immunological risks of systemic caspase-8 blockade (55, 56). These phenotypes also align with the role of caspase-8 in restraining necroptotic signaling and maintaining immune homeostasis.

Consequently, a more viable strategy involves “fine-tuning” the pathway rather than implementing a total, indiscriminate blockade of key molecules. Moreover, the timing and site of intervention must be strictly regulated—ideally, therapeutic efforts should inhibit PANoptosis locally at the lesion site during the acute phase of injury, thereby avoiding long-term systemic immune suppression(Details on existing studies targeting PANoptosis interventions are shown in **Table 1**).

**Table 1:** Therapeutic strategies targeting PANoptosis after SCI.

Key factor	Treatments	Experimental models	Methods of administration	Therapeutic effects	Reference
TRIM56	Slowly microinjected at the site of spinal cord injury(AAVs).	Spinal cord contusion surgery	TRIM56/YBX1/ZBP1	YBX1 stabilizes Zbp1 mRNA, thereby promoting ZBP1 expression. TRIM56, functioning as an E3 ubiquitin ligase, modulates ZBP1-mediated PANoptosis by regulating the ubiquitination and degradation of YBX1.	(11)
Lgals3	Intraperitoneal injection of ZnG at 30 mg/kg	A moderate spinal cord contusion model was established in mice by performing a T9/T10 laminectomy followed by a 50 K dyn force impact using a spinal cord impactor.	Lgals3/Bax/PANoptosis	Regulation via the Lgals3–Bax axis stabilizes mitochondrial quality, thereby inhibiting neuronal PANoptosis.	(57)
ZBP1	Baicalin	Primary bone marrow-derived macrophages (BMDMs) and the J774A.1 macrophage cell line (*in vitro*)	Mitochondrial Z-DNA formation/ZBP1-PANoptosome	Baicalin inhibits PANoptosis in macrophages by blocking mitochondrial Z-DNA formation and ZBP1–PANoptosome assembly, thus conferring protection against inflammatory diseases.	(58)
ROS	Scutellarin	PANoptosis was induced in murine macrophages (BMDMs and J774A.1 cells) using the TAK1 inhibitor 5Z-7-oxozeaenol (OXO) combined with LPS stimulation.	ROS/PANoptosis	Scutellarin inhibits PANoptosis by blocking the assembly of the PANoptosome (an ASC/RIPK3/Caspase-8/ZBP1/p-MLKL complex) and reducing mitochondrial ROS generation, thereby preserving mitochondrial function	(59)
The canonical pathways of pyroptosis, apoptosis, and necroptosis.	Melatonin	In the rats of the SCIR group, the abdominal aorta was blocked below the right renal artery near the heart using a 50 g aneurysm clip for 60 min.	–	Melatonin acted as an anti-PANoptotic agent, preventing the loss of spinal cord neurons and motor function; it exhibited anti-apoptotic, anti-necroptotic, and anti-pyroptotic effects.	(44)
The canonical pathways of pyroptosis, apoptosis, and necroptosis.	Intraperitoneal injection of GYY4137 (a slow-releasing H2S donor)	The abdominal aorta was blocked below the right renal artery near the heart using a 50 g aneurysm clip for 60 min.	The slow-releasing H_2_S donor GYY4137 improved neurological function and neuronal preservation after SCIRI	GYY4137 alleviated SCIRI by dampening microglia/macrophage-driven neuroinflammation while simultaneously inhibiting neuronal pyroptosis, apoptosis, and necroptosis (PANoptosis).	(60)

One logical therapeutic angle is to inhibit crucial components of the PANoptosome or their upstream regulators. For example, ZBP1 – being a pivotal initiator of PANoptosis in neurons – is an attractive target. Direct inhibitors of ZBP1 are not yet available, but strategies that reduce its expression or activation can mitigate PANoptosis. Adeno-associated virus (AAV)-mediated delivery of an shRNA against Zbp1 in mice effectively knocked down ZBP1 in the spinal cord and significantly reduced PANoptotic cell death. This treatment led to improved histological and functional outcomes after SCI (11). Another target in the same pathway is YBX1, the mRNA stabilizer of Zbp1. Although no specific YBX1 inhibitors are clinically available, the identification of TRIM56 as a natural suppressor of YBX1 suggests a therapeutic approach. Enhancing TRIM56 activity or expression could in turn promote YBX1 degradation and limit PANoptosis. Small molecules that upregulate TRIM56 or mimic its E3 ligase function might thus serve as PANoptosis inhibitors. In inflammatory disease models, other PANoptosome components like caspase-8 and RIPK3 have been targeted by existing inhibitors (e.g., the pan-caspase inhibitor emricasan and RIPK1/RIPK3 inhibitors such as necrostatins) (61, 62). Although these inhibitors, originally designed to block a single mode of cell death, have begun to be used in PANoptosis research, they have not yet been applied in SCI models. We speculate that targeting PANoptosis in the context of SCI could yield synergistic protective effects.

Mitochondria are central players in apoptosis (they release cytochrome c to trigger caspase activation) and also contribute to pyroptosis and necroptosis by releasing DAMPs such as mitochondrial DNA and ROS. Thus, protecting mitochondrial integrity after SCI can indirectly suppress PANoptosis (63). A recent study found that in SCI model animals treated with zinc, mitochondrial quality control functions improved (e.g. upregulation of mitophagy and antioxidant defenses), and the preservation of mitochondria significantly reduced PANoptosis in the spinal cord (57). Zinc treatment inhibited the Lgals3–Bax pathway, which is associated with outer mitochondrial membrane permeabilization and initiation of apoptosis. By preventing activation of the pro-apoptotic protein Bax and the release of mitochondrial contents, zinc reduced both apoptotic and pyroptotic cell death. Baicalin is a flavonoid derived from *Scutellaria baicalensis (Chinese skullcap)*. It has known anti-inflammatory and anti-oxidative effects, and notably it has been found to inhibit various forms of regulated cell death (64, 65). Baicalin treatment dose-dependently inhibited this cell death and was shown to prevent mitochondrial injury and subsequent mtDNA release and Z-DNA formation, thereby impairing the recruitment of ZBP1, RIPK3, ASC, and caspase-8 into the PANoptosome (58). Essentially, baicalin kept the mitochondria intact and the DNA in the proper conformation, removing the trigger for PANoptosome assembly. Scutellarin is a flavonoid isolated from herbal Erigeron breviscapus Hand. Scutellarin was shown to inhibit PANoptotic cell death in macrophages by suppressing the activation of all key markers of the three pathways (64). This included pyroptosis indicators (caspase-1 p10 fragment and GSDMD-N terminus), apoptosis indicators (cleaved caspase-3/8/9 and GSDME-N terminus), and a necroptosis indicator (phosphorylated MLKL) (59). It prevented the assembly of a PANoptosome complex containing ASC, RIPK3, caspase-8, and ZBP1, suggesting that its action occurs at an early upstream stage of PANoptotic signaling and does not depend on NLRP3 or caspase-1. This highlights scutellarin’s notable potential to mitigate PANoptotic damage by reducing mitochondrial injury and lowering mitochondrial ROS levels in macrophages.

Additionally, numerous drugs with long histories of clinical use have demonstrated potential in suppressing PANoptosis after SCI, thereby revealing new therapeutic targets. These compounds often have pleiotropic actions, targeting oxidative stress, inflammation, and cell death simultaneously – an ideal profile for countering PANoptosis. Melatonin, a neurohormone with potent antioxidant properties, was tested in a rat model of spinal cord ischemia-reperfusion (SCIR) injury (66, 67). A recent study found that melatonin administration after SCIR not only improved motor function but also selectively reduced markers of apoptosis, necroptosis, and pyroptosis in the spinal cord (44). Specifically, melatonin treatment lowered the number of TUNEL-positive (apoptotic) neurons, decreased the levels of phosphorylated MLKL (necroptotic marker), and reduced caspase-1 activation and IL-1β release (pyroptotic indicators) compared to untreated injured controls. Meanwhile, studies have found that hydrogen sulfide (H_2_S) can also promote recovery after SCI. GYY4137 (a slow-releasing H_2_S donor) treatment attenuated the number of TUNEL-positive and cleaved caspase-3-positive cells was decreased, and the upregulation of expression of cleaved caspase-8, cleaved caspase-3, Bax, and Bad and downregulation of Bcl-2 expression were reversed after GYY4137 administration (60). This suggests that while pursuing new targets, we should not overlook drugs with broad effects on multiple cell death pathways, as such agents might hold a key to modulating PANoptosis and improving recovery in SCI.

## 5 Discussion and future directions

Viewing secondary cell death after SCI as an integrated process of PANoptosis offers a novel perspective for understanding secondary injury. Traditional neuroprotective strategies for SCI have mostly focused on single pathways, targeting apoptosis, pyroptosis, or neuroinflammation in isolation (68, 69). The emergence of the PANoptosis concept indicates that these cell death pathways are interrelated, such that blocking one in isolation is often insufficient and may even be counterproductive. By contrast, targeting PANoptosis means intervening at points of convergence or common upstream triggers, thereby suppressing the entire pro-inflammatory cell death network. This holistic approach is especially important for SCI, as the injury triggers a multitude of concurrent biochemical insults that activate multiple death programs almost simultaneously (70, 71). Disrupting the self-amplifying cycle between inflammation and cell death is expected to halt the progression of tissue damage and create a more favorable environment for regeneration and repair.

SCI triggers multiple concurrent RCD pathways. Pyroptosis, apoptosis, and necroptosis – the so-called PANoptosis triad – have been observed in injured spinal tissue alongside non-canonical RCD forms like ferroptosis, parthanatos, and autophagy-dependent cell death. Emerging evidence indicates extensive crosstalk among these pathways (72). Enhancing autophagy flux after SCI was shown to concurrently suppress pyroptotic and necroptotic injury, presumably by accelerating the clearance of inflammasomes and necroptosis effectors (73). Similarly, targeting shared mediators can dampen multiple death programs: inhibiting the FOS/USP53 signaling axis promotes ubiquitin–proteasome degradation of gasdermin D and MLKL, thereby attenuating both pyroptosis and necroptosis in injured neurons (74). Likewise, iron-catalyzed lipid peroxidation (ferroptosis) co-occurs with inflammatory cell death in the lesion; notably, microglia undergoing apoptosis and pyroptosis in acute SCI also exhibit a surge of ferroptotic death in the early post-injury phase (72). Collectively, these findings illustrate that PANoptosis and non-canonical RCD pathways are mechanistically intertwined after SCI, and targeting one death modality can influence others, underscoring the need for multi-modal therapeutic strategies.

One key issue for future research is to determine the optimal intervention targets within the PANoptosis pathway. Targeting core molecules such as caspase-8 or ZBP1 could provide broad control of PANoptosis. However, this strategy may also carry risks, as these proteins play physiological roles in host defense and tissue homeostasis. The discovery of the TRIM56–YBX1–ZBP1 axis provides a concrete targeting mechanism (11). Enhancing TRIM56 activity or intervening in YBX1 can reduce the upregulation of ZBP1, thereby producing an inhibitory effect on PANoptosis. Although these strategies are still in early stages, they have opened a pathway to designing therapies that selectively reduce PANoptosis in injured neurons.

From a clinical perspective, one exciting prospect is the repurposing of drugs with established safety profiles as PANoptosis inhibitors in SCI. Drugs already used in clinical practice—including melatonin, H_2_S donors, baicalin, and scutellarin—have demonstrated potential to interfere with PANoptosis in SCI models (44, 58–60). This implies that such agents could move into clinical trials more quickly and safely, lending greater practical significance to PANoptosis-targeted therapy.

Another future direction is to seek PANoptosis biomarkers to guide SCI treatment. If we can detect the occurrence of PANoptosis in a patient’s blood or cerebrospinal fluid—for example, by measuring a panel that captures pyroptotic (caspase-1, IL-1β/IL-18), apoptotic (cleaved cytokeratin-18 or caspase-cleaved substrates), and necrotic/necroptotic (HMGB1 release, phosphorylated MLKL-related signatures) components—then we may be able to stratify patients for PANoptosis-targeted interventions. In practice, such biomarker panels could be assessed using ELISA or multiplex immunoassays, targeted proteomics, or emerging single-cell and spatial transcriptomic approaches on clinical samples. These methods would enable longitudinal monitoring of inflammatory cell death dynamics across acute and subacute phases.

One concern is that indiscriminate and prolonged suppression of PANoptosis might inadvertently blunt inflammatory programs that contribute to debris clearance and tissue remodeling. Notably, recent evidence indicates that pyroptotic cells can release lipid mediators and metabolites that promote tissue repair in certain contexts, suggesting that inflammatory cell death may exert context-dependent pro-repair effects (75). Therefore, in SCI the therapeutic goal may be time- and site-restricted modulation of PANoptosis during the acute secondary-injury phase, rather than complete systemic blockade. We must consider whether interventions targeting PANoptosis might disrupt normal recovery processes during the healing phase. It may be more advantageous to further study the post-SCI time window of programmed cell death and to modulate PANoptosis for a partial inhibition or delayed intervention, rather than attempting complete suppression from the outset.

Moreover, the timing and site of intervention must be strictly regulated. Ideally, therapeutic efforts should inhibit PANoptosis locally at the lesion site during the acute phase of injury. This targeted approach helps avoid long-term systemic immune suppression. From a translational standpoint, this requirement motivates the development of spatiotemporally controlled, lesion-confined delivery platforms. Lesion-targeting nanocarriers can be designed to improve drug accumulation within the injured spinal cord while reducing peripheral exposure by combining blood–spinal cord barrier (BSCB)-penetrating elements and injury-microenvironment homing ligands that bind lesion-associated extracellular matrix components or activated neuroglial/immune cell populations (76). In parallel, hyaluronic acid (HA)-based nanoparticles can leverage receptor-mediated interactions (e.g., CD44-mediated lesion enrichment or glial uptake) to concentrate antioxidant and anti-inflammatory payloads at the injury site (77). This targeted delivery attenuates secondary injury cascades and supports neurological recovery. Notably, an injectable, photocurable lipid nanoparticle–GelMA (“PLNG”) hydrogel scaffold has been used for localized siRNA delivery with controlled release and depot degradation on approximately week-scale kinetics, producing multi-cell-type immunomodulatory effects and promoting axonal regeneration after SCI (78). Beyond single-agent delivery, scaffold-based depots (implantable or in situ-forming) enable co-delivery of multi-modal therapeutics and provide 3D structural cues that reshape the lesion microenvironment. For instance, photocurable scaffolds have been engineered for co-delivery of MIF-targeted siRNA and GDNF protein. This strategy links local inflammatory reprogramming with pro-regenerative neurotrophic signaling, and it improved tissue repair and motor recovery in preclinical SCI models (79).

In summary, examining post-SCI cell death through the overarching concept of PANoptosis is highly significant. This perspective emphasizes the importance of systemic intervention in addressing complex CNS injuries — not just targeting a single molecule, but the entire network. Through continued research into this mechanism, we hope to translate these insights into clinically feasible therapeutic strategies. Such advances could help to reduce the lifelong disability burden of SCI.
